# Probability-Based Estimates of Severe Acute Respiratory Syndrome Coronavirus 2 Seroprevalence and Detection Fraction, Utah, USA

**DOI:** 10.3201/eid2711.204435

**Published:** 2021-11

**Authors:** Matthew H. Samore, Adam Looney, Brian Orleans, Tom Greene, Nathan Seegert, Julio C. Delgado, Angela Presson, Chong Zhang, Jian Ying, Yue Zhang, Jincheng Shen, Patricia Slev, Maclean Gaulin, Mu-Jeung Yang, Andrew T. Pavia, Stephen C. Alder

**Affiliations:** Veterans Affairs Salt Lake City Health Care System, Salt Lake City, Utah, USA (M.H. Samore);; University of Utah, Salt Lake City (M.H. Samore, A. Looney, B. Orleans, T. Greene, N. Seegert, J.C. Delgago, A. Presson, C. Zhang, J. Ying, Y. Zhang, J. Shen, P. Slev, M. Gaulin, M.-J. Yang, A.T. Pavia, S.C. Alder)

**Keywords:** respiratory infections, severe acute respiratory syndrome coronavirus 2, SARS-CoV-2, SARS, COVID-19, coronavirus disease, zoonoses, viruses, coronavirus, antibodies, case detection, IgG, immunoglobulin G, incidence, infections, nasopharyngeal swabs, PCR, population surveillance, probability sampling design, reverse transcription PCR, rRT-PCR, sensitivity, specificity, seroepidemiologic studies, serology, seroprevalence, Utah, United States

## Abstract

We aimed to generate an unbiased estimate of the incidence of severe acute respiratory syndrome coronavirus 2 (SARS-CoV-2) infection in 4 urban counties in Utah, USA. We used a multistage sampling design to randomly select community-representative participants >12 years of age. During May 4–June 30, 2020, we collected serum samples and survey responses from 8,108 persons belonging to 5,125 households. We used a qualitative chemiluminescent microparticle immunoassay to detect SARS-CoV-2 IgG in serum samples. We estimated the overall seroprevalence to be 0.8%. The estimated seroprevalence-to-case count ratio was 2.5, corresponding to a detection fraction of 40%. Only 0.2% of participants from whom we collected nasopharyngeal swab samples had SARS-CoV-2–positive reverse transcription PCR results. SARS-CoV-2 antibody prevalence during the study was low, and prevalence of PCR-positive cases was even lower. The comparatively high SARS-CoV-2 detection rate (40%) demonstrates the effectiveness of Utah’s testing strategy and public health response.

By May 2021, >150 million severe acute respiratory syndrome coronavirus 2 (SARS-CoV-2) infections and >3 million deaths from coronavirus disease (COVID-19) had been reported worldwide (*1*). The real infection count likely is much higher but continues to be a point of uncertainty. Case reporting underestimates the total number of SARS-CoV-2 infections because of underdetection of asymptomatic or mildly symptomatic cases and variation in the use and availability of diagnostic testing. Serologic testing provides an independent method to estimate the true cumulative incidence of SARS-CoV-2 infection because it relies on evidence of immune response as an indication of previous infection. Seroprevalence has been touted as a more standardized way to estimate the incidence of SARS-CoV-2 infection across different populations, but inconsistencies in test performance and sampling methods continue to cause challenges for use of seroprevalence.

In May 2020, the University of Utah (Salt Lake City, Utah, USA) launched the Utah Health and Economic Recovery Outreach project, in partnership with state government agencies, to collect community-based data on SARS-CoV-2 infection rates. Our goal was to estimate the cumulative incidence of SARS-CoV-2 infection to benchmark case detection in community populations based on public health surveillance. In addition to measuring SARS-CoV-2 seroprevalence, we collected nasopharyngeal swab samples to concurrently estimate the prevalence of reverse transcription PCR (RT-PCR) positivity. We applied methods of recruitment and analysis to minimize bias and maximize relevance for policymaking. We describe the results of the first phase of the project, which was conducted in the Wasatch Front, the major population center of Utah, comprising a chain of contiguous cities and towns stretched along the Wasatch Mountain Range.

## Methods

### Sampling Design and Participant Recruitment

We conducted serologic survey in 4 counties: Utah, Salt Lake, Davis, and Summit. The total estimated population of the study area is ≈2.2 million, which represents ≈68% of the population of Utah. Overall, 29% of the population is <18 years of age, compared with 22% of the US population (*2*). The fraction of residents of the 4 counties that are non-Hispanic White is 76%, which is higher than the US population of 60%. During March 14–June 30, 2020, the 4 counties reported 17,316 cases of SARS-CoV-2 infection (*3*).

We recruited and enrolled participants during May 4–June 30, 2020. The sampling frame consisted of a list of all residential addresses in the 4 counties curated by the state of Utah. The 657,870 total addresses were grouped hierarchically into 16,677 census blocks, 1,089 census block groups, 389 census tracts, and 229 groups of adjacent tracts, termed tract groups. We categorized tract groups into 15 strata based on combinations of county, ethnicity, median age, and reported positive case count from the Utah Department of Health.

We used 2 address-based probability sampling designs that differed in intensity of recruitment and geographic clustering. Both methods followed a random sampling design. Our primary sampling design included 11,563 addresses that were selected by randomly choosing 26 of the tract groups from the 15 strata, weighted by tract group population. We then selected ≈420 addresses from each tract group by first randomly choosing 30 census block groups per census tract group and then selecting 14 addresses per census block group. The geographic address clustering facilitated recruitment and data collection and followed methods recommended by the Centers for Disease Control and Prevention (https://www.cdc.gov/nceh/casper/sampling-methodology.htm).

Our secondary sampling frame comprised 14,012 addresses. We selected these addresses by proportionately oversampling the same strata as our primary sampling frame and excluding the tract groups selected in our primary sampling frame. The secondary sampling frame enabled us to expand the pool of participants and to broaden the geographic reach within the 4 counties.

To recruit our sample, we sent each address a postcard and a letter encouraging household members to participate. Participants were asked to complete a household survey, and household members >12 years of age were invited to take an individual participant survey and to undergo testing for IgG and viral RT-PCR at a specified mobile testing site. In our primary sampling frame, home addresses also were visited by a recruitment field team that attempted <3 in-person contacts. All household members who completed the survey and were tested received a $10 gift card as compensation for their time.

Each mobile testing site location included 4 sequential drive-through stations. The first station collected basic information about the persons in the vehicle; the second conducted the viral RT-PCR sample via nasopharyngeal swab; the third conducted the IgG test via blood draw; and the last quality-checked participation, provided information about receiving test results, and responded to participant questions. The analyses described here are limited to persons who completed the participant survey and underwent serologic testing.

### Laboratory Methods

We analyzed serum specimens by using the SARS-CoV-2 IgG assay (Abbott Laboratories, https://www.abbott.com) on an Architect i2000 instrument (Abbott Laboratories), according to the manufacturer’s instructions. The SARS-CoV-2 IgG assay is a qualitative chemiluminescent microparticle immunoassay that detects IgG binding to an undisclosed epitope of the SARS-CoV-2 nucleocapsid protein. The assay relies on an assay-specific calibrator to report a ratio of specimen absorbance to calibrator absorbance. The assay can be interpreted as positive (ratio >1.4) or negative (ratio <1.4). The manufacturer reports a sensitivity of 86.4% (95% CI 65.1%–97.1%) 8–13 days after symptom onset and 100% (95% CI 95.9%–100%) >14 days after symptom onset, and a specificity of 99.6% (95% CI 99.1%–99.9%) (*4*,*5*). The manufacturer’s estimate of sensitivity >14 days after symptom onset was derived from 88 symptomatic patients. However, other studies using this assay have reported lower sensitivities, ranging from 85% to 97%, when used in the general population (*6*–*8*). We observed that 20/24 (83.3%) participants who reported a prior positive SARS-COV-2 test >14 days before we collected serum samples were seropositive. By using a cutoff of 10 days after a prior positive SARS-COV-2, 25/30 (83.3%) participants who reported prior positive tests also were IgG positive. Therefore, we assumed a sensitivity of 83% in our primary analysis.

We used the cobas SARS-CoV-2 assay (Roche Diagnostics, https://www.roche.com) to detect viral RNA in nasopharyngeal swabs, according to the manufacturer’s instructions. The cobas SARS-CoV-2 assay detects the nonstructural open reading frame (ORF) 1a/b region unique to SARS-CoV-2 at a limit of detection of 1,800 copies/mL. All testing was performed at ARUP Laboratories (https://www.aruplab.com), a nonprofit national reference laboratory associated with the University of Utah. 

For data analysis, we used a series of steps to account for the sampling design, nonresponse, demographic balance, and the sensitivity and specificity of the serology assay. The University of Utah Institutional Review Board designated this surveillance project nonresearch because it was launched to support public health and governmental response to the COVID-19 pandemic. 

### Statistical Methods

#### Sampling Design

We computed sampling design weights to account for varying probabilities of sampling of households (Appendix) (*9*). These weights depended primarily on the ratios of the numbers of sampled households to the total numbers of households within each stratum of the primary and secondary sampling designs (Appendix Tables 1–6). We computed 3 further sets of weights to account for nonresponse at the household, participant, and serology testing levels. We determined household response weights from estimated propensities of household response based on characteristics of the census block group where the household was located and participant response weights from estimated propensities of response by persons within households based on characteristics of the census block group and the primary household respondent. We determined serology response weights from estimated propensities for the provision of a serology sample based on participant survey responses. 

We estimated propensities separately in the primary and secondary sampling designs by using nonparametric boosted regression for household and serology response and logistic regression for participant response (Appendix Table 1) (*10*). We used estimated propensities for membership in the primary versus the secondary design to align the secondary sampling design’s characteristics to those of the primary sampling design. Multiplication of each of the described weights provided 2 sets of comprehensive weights that accounted for the design and nonresponse for the primary and secondary sampling designs. We then scaled the weights for 2 sampling designs based on the proportions of respondents in the 2 designs to provide a single final set of weights for estimating seroprevalence across the 4-county area. To prevent extreme variation in weights, we truncated weights that were either <10% or >10-fold greater than the median weight. Finally, we used iterative proportional fitting to optimize agreement of the marginal distributions of age, sex, Hispanic ethnicity, and education level between the weighted study sample and the US census data for the 4-county area (*11*).

#### Data Analysis

We defined the primary sampling units (PSUs) for data analysis by 54 census tracts included in the primary sampling design and mainly by block groups in the secondary sampling design. For Summit County, sampling was performed without clustering at the household level in the secondary sampling frame, so the household served as the PSU. We modeled the relationship of seroprevalence to predictor variables, such as county, demographic and clinical factors, behaviors, and attitudes, by using survey weighted generalized linear models for binary outcomes and assessed variability based on replicate jackknife weights (*12*,*13*). We tested for the presence of a detectable temporal trend in seroprevalence by including calendar time as a continuous variable in models relating seroprevalence to the Utah Department of Health case count. These analyses showed no trend for an effect of calendar time. Hence, we performed analyses for seroprevalence without adjustment for calendar time.

We corrected estimates of seroprevalence for assay error by applying the following formula (Figure): 

**Figure:**
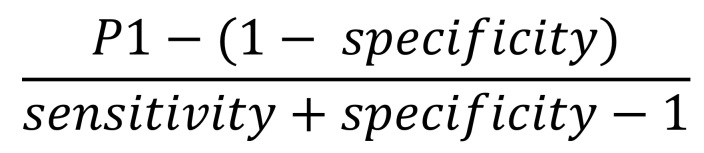
We corrected estimates of seroprevalence for assay error by applying the following formula, where P1 is the estimated prevalence within a given category of a predictor variable provided by the generalized linear models.

where P1 is the estimated prevalence within a given category of a predictor variable provided by the generalized linear models. We then used the parametric bootstrap to account for the sampling error and 95% CI of the manufacturer’s estimate of specificity. We estimated the seroprevalence-to-case-count ratio by computing the ratio between the adjusted prevalence estimates we described in the previous section to the weighted average case count rates corresponding to the respondent’s ZIP code 10–17 days before the respondent’s serology test reported by the Utah Department of Health. The inverse of the ratio of adjusted prevalence and average case counts is the detection fraction, the estimated proportion of the total number of infections that were reported. We performed hypothesis tests comparing prevalence between categories directly on the estimates of seroprevalence without assay error adjustment because assay error adjustment does not affect equality of seroprevalences between subgroups when sensitivity plus specificity is >1.

## Results

### Participant Characteristics

During May 4–June 30, 2020, we randomly selected 11,563 households for a combined mailed recruitment and home visit and randomly selected another 14,012 households for mailed recruitment only. Altogether, 8,108 persons from 5,125 households completed surveys and testing for SARS-CoV-2 antibodies. Among participants, 5,791 were in the combined home visit and mailed recruitment frame and 2,317 were in the mailed recruitment only frame. The median age of participants was 44 (interquartile range [IQR] 30–62) years; only 9.3% of participants were 12–18 years of age (Tables 1, 2). Overall, 6.6% of participants self-reported ethnicity as Hispanic, compared with 15.3% of the 4-county population based on census data. The source population also differed from participants with respect to age distribution and education level. Accounting for response bias through iterative proportional fitting resolved these differences in county-level marginal distributions.

**Table 1:** Characteristics of participants and households in a study of SARS-CoV-2 seroprevalence, Utah, United States

Household-level factors	No. (%) participating households	No. (%) participants, n = 8,108
County	n = 5,125	
Davis	1,023 (20)	1,703 (21.0)
Salt Lake	2,695 (52.6)	4,021 (49.6)
Summit, including Park City	283 (5.5)	345 (4.3)
Utah	1,124 (21.9)	2,039 (25.1)
No. participating household members	n = 5,088	
1	1,738 (34.2)	1,027 (12.7)
2	2,277 (44.8)	3,683 (45.4)
3	541 (10.6)	1,307 (16.1)
>4	532 (10.5)	2,091 (25.8)
No. household members <12 years of age	n = 5,033	
0	3,537 (70.3)	5,407 (67.6)
1	589 (11.7)	1,053 (13.2)
2	499 (9.9)	850 (10.6)
3	239 (4.7)	424 (5.3)
>4	169 (3.4)	269 (3.4)
Primary language spoken in household	n = 5,053	
English	4,866 (96.3)	7,785 (97.1)
Spanish	132 (2.6)	169 (2.1)
Other	55 (1.1)	61 (0.8)

*Participants completed a survey and had serum collected to test for SARS-CoV-2 IgG. n values indicate number of responses available in that category. SARS-CoV-2, severe acute respiratory syndrome coronavirus 2.

**Table 2:** Characteristics of participants in a study of SARS-CoV-2 seroprevalence, Utah, USA*

Characteristics	No. (%) participants, n = 8,108
Sex	
F	4,335 (53.5)
M	3,773 (46.5)
Age, y	
12–<18	755 (9.3)
18–<45	3,366 (41.5)
45–64	2,345 (28.9)
65–74	1,087 (13.4)
>75	555 (6.8)
Ethnicity, n = 8044	
Hispanic	528 (6.6)
Non-Hispanic	7,516 (93.4)
Race, n = 7,839	
White	7,452 (95.1)
Black or African American	34 (0.4)
American Indian or Alaska Native	32 (0.4)
Asian	159 (2.0)
Native Hawaiian or other Pacific Islander	40 (0.5)
Multiracial	122 (1.6)
Underlying conditions	
Diabetes	508 (6.3)
Hypertension	1,078 (13.3)
Cardiovascular disease	354 (4.4)
Asthma	841 (10.4)
Emphysema	72 (0.9)
Cancer	130 (1.6)
Immunosuppressive therapy	79 (1.0)
Exposure, n = 8,084	
Contact with COVID-19 case	360 (4.5)
Prior testing	
Tested for COVID-19 at any time	716 (8.8)

*Participants completed survey and had serum collected to test for SARS-CoV-2 IgG. n values indicate number of responses available in that category COVID-19, coronavirus disease; SARS-CoV-2, severe acute respiratory syndrome coronavirus 2.

### Estimated Seroprevalence

Among participants, 89 persons from 75 households were seropositive, corresponding to an unadjusted seroprevalence of 1.1% (Table 3). The 4-county seroprevalence adjusted for sampling fraction, nonresponse, and test performance was 0.8% (95% CI 0.1%–1.6%). We estimated adjusted SARS-CoV-2 seroprevalence to be 5.7% (95% CI 1.2%–19.4%) among persons residing in households where the primary language was Spanish and 2.7% (95% CI 0.6%–8.0%) among persons who self-reported as Hispanic; both estimates were significantly greater than the comparator groups (p = 0.01 for Spanish as primary language; p = 0.03 for self-report as Hispanic) (Table 3). Seroprevalence was 4.6% in Summit County, which includes the ski resort town, Park City, an early infection hot spot in Utah, and was significantly higher than the other counties (p = 0.03); the variation in seroprevalence across Utah, Salt Lake, and Davis counties was not statistically different.

**Table 3:** Overall and subgroup-specific seroprevalence of participants in a study of SARS-CoV-2 seroprevalence, Utah, USA*

Characteristics	Total	No. (%) seropositive	Adjusted seroprevalence, % (95% CI)†	p value
Overall	8,108	89 (1.1)	0.8 (0.1–1.6)	
County				
Davis	1,703	16 (0.9)	0.1 (0–1.3)	0.06
Salt Lake	4,021	38 (0.9)	0.7 (0–1.8)	
Summit, including Park City	345	10 (2.9)	4.6 (1.0–15.1)	
Utah	2,039	25 (1.2)	1.2 (0.1–3.4)	
Sex				
M	3,773	41 (1.1)	0.7 (0–1.6)	0.65
F	4,293	48 (1.1)	0.9 (0.2–1.9)	
Age, y				
<45	4,119	39 (0.9)	0.9 (0.1–2.1)	0.62
45–64	2,345	31 (1.3)	0.8 (0.1–1.7)	
>65	1,642	19 (1.2)	0.4 (0–1.4)	
Ethnicity				
Non-Hispanic	7,516	75 (1)	0.5 (0–1.1)	0.03
Hispanic	528	14 (2.7)	2.7 (0.6–8.0)	
Primary language spoken in household				
English	7,785	78 (1)	0.5 (0–1.2)	0.01
Spanish	169	11 (6.5)	5.7 (1.2–19.4)	
No. participants in household				
1	1,027	15 (1.5)	0.7 (0–1.8)	0.60
2	3,683	35 (1)	0.5 (0–1.7)	
>3	3,398	39 (1.1)	1.0 (0.2–2.3)	
No. participants <12 years of age				
0	5,407	64 (1.2)	0.6 (0–1.3)	0.33
>1	2,596	20 (0.8)	1.1 (0.1–3)	
Cumulative incidence per 100,000 residents in participant’s ZIP code				
<200	3,718	26 (0.7)	0.2 (0–0.9)	0.02
200–500	3,012	34 (1.1)	0.8 (0.1–2.0)	
>500	1,378	29 (2.1)	2.2 (0.6–5.5)	

*Participants completed survey and had serum collected to test for SARS-CoV-2 IgG. COVID-19, coronavirus disease; SARS-CoV-2, severe acute respiratory syndrome coronavirus 2. †Adjusted for sampling design and test sensitivity (0.83) and specificity (0.996)

Seroprevalence correlated with cumulative incidence estimated on the basis of reported case counts (Table 3). The adjusted seroprevalence was 2.2% in ZIP codes where cumulative incidence calculated from reported cases was >500/100,000 population compared with 0.2% in ZIP codes in where the reported cumulative incidence was <200/100,000 population. The overall seroprevalence-to-case count ratio was estimated to be 2.5 (95% CI 0.3–5.0), corresponding to a detected fraction of 0.40. This ratio was not statistically different across the 4 counties.

### Other Descriptive Analyses

Among participants, 360 (4.4%) reported contact with a person with diagnosed COVID-19 and 26 (7.2%) of these participants were seropositive (Table 4). Among participants who reported contact with a family member with known SARS-CoV-2 infection, 14.4% were seropositive. In contrast, among 38 persons who reported exposure to SARS-CoV-2 infection in their role as healthcare workers, none were seropositive. Among 62 households with >2 members who tested positive, our analysis revealed 53 households with exactly 1 seropositive member and 9 households with >1 seropositive member. Among the 123 members of 62 households with SARS-CoV-2–positive residents, 23 (18.7%) participants were seropositive. We assumed that infection for 1 of the infected members of each household was imported and that other household cases were transmissions from the index member of the household; thus, our crude estimate the secondary household attack rate was 12%.

**Table 4:** Relationship between COVID-19 exposures and serologic results of participants in a study of SARS-CoV-2 seroprevalence, Utah, USA*

Exposures	Total	No. (%) seronegative, n = 8,019	No. (%) seropositive, n = 89	% Adjusted seroprevalence (95% CI)†
Contact with diagnosed COVID-19 case	360	334 (92.8)	26 (7.2)	8.5 (3.3–19.5)
Participant’s relationship with contact				
Family member	97	83 (85.6)	14 (14.4)	14.8 (4.0–40.8)
Friend	42	38 (90.5)	4 (9.5)	14.0
Healthcare worker‡	38	38 (100)	0 (0)	0.0
Coworker	105	102 (97.1)	3 (2.9)	3.4
Other	78	73 (93.6)	5 (6.4)	3.1 (0.3–12.9)
Reside in household with >1 seropositive person	123	100 (81.3)	23 (18.7)	24.9 (10.5–48.7)

*COVID-19, coronavirus disease; SARS-CoV-2, severe acute respiratory syndrome coronavirus 2. †Adjusted for sampling design and test sensitivity (0.83) and specificity (0.996). Confidence intervals are omitted for subgroups with fewer than 5 seropositive persons. ‡Participant reported that their exposure was related to their work as a healthcare worker.

Overall, 798 (9.9%) persons reported having a prior COVID-19 test. Among 30 participants who reported having a positive COVID-19 test >14 days before serum collection, 25 (83.3%) were SARS-CoV-2 seropositive; we used that figure to estimate the sensitivity of the serologic assay. Among seropositive participants, 7 (28.0%) reported a prior RT-PCR–positive SARS-CoV-2 test. If we assume a true seroprevalence of 0.8%, assay sensitivity of 83%, and specificity of 99.6%, the corrected point estimate for the detection fraction based on history of a prior positive RT-PCR test is 0.28/0.614 = 0.46, which is close to our estimated detection fraction based on the seroprevalence-to-case count ratio.

Among 6,251 participants from whom a nasopharyngeal swab specimen was collected, 14 (0.2%) had SARS-CoV-2 virus detected by RT-PCR; 9 (64.3%) of those persons were seropositive. The small number of positive RT-PCR tests precluded statistical analysis of factors associated with positivity or adjustment for response bias.

## Discussion

By using a statistical sampling frame and adjusting for test performance and non-response, we estimated the prevalence of IgG to SARS-CoV-2 in 4 urban counties in Utah during May–June 2020 to be only 0.8%. Thus, consistent with other community surveys, most of the population lacked immunity to SARS-CoV-2. Comparing seroprevalence to the cumulative incidence of SARS-CoV-2 infection based on case reporting, we found that the estimated ratio of total-to-detected cases was 2.5, corresponding to a detection fraction of 40%. We found participants in Summit County had higher seroprevalence of 4.6%, which is compatible with the extensive outbreak in the resort community of Park City that began in March 2020. Seroprevalence was higher (2.7%) among persons who identified as Hispanic than among those who identified as non-Hispanic (0.5%); seroprevalence was 5.7% among persons who lived in a household where Spanish was the primary language, much higher than the 0.5% seroprevalence among persons who lived in households where English was the primary language. This finding adds to the substantial body of evidence regarding ethnic and racial disparities in the spread of SARS-CoV-2 across populations.

Our estimates of seroprevalence and of the seroprevalence-to-case count ratio are generally lower than has been reported in Utah and elsewhere in the United States during a similar time. Several seroprevalence studies conducted in the United States and other countries have been published (*14*–*24*) and use a variety of assays and sampling methods (*25*). Some studies have relied on convenience samples or did not adequately control for response bias. The specificity of serologic methods for SARS-CoV-2 testing varies widely, which can lead to substantial overestimation in a low-prevalence population (*26*). Not all studies have adjusted for test performance, and the differences in methods make comparisons between studies challenging.

Our project involved random sampling of >25,000 households and used intensive recruiting methods. Our analytical approach accounted for multiple sources of error, including response bias and imperfect test performance. We also were able to generate an internal estimate of the detection fraction by using self-reported histories of prior RT-PCR test results. After accounting for test error, the estimate of the detection fraction based on participant histories was 0.46, a value that corroborates our population estimate of the detected fraction of 0.40.

We used a serologic test that is reported by the manufacturer to have a specificity at 99.6% (*4*,*5*); however, even at this level of accuracy, statistically accounting for false positive results is necessary given the low population prevalence of IgG to SARS-CoV-2. To better account for the possibility of reduced sensitivity when asymptomatic infections are included (*27*), we assumed a sensitivity of 83% because of an analysis of project participants who reported having had a positive RT-PCR test in the past. We note that our estimate of sensitivity is substantially lower than the manufacturer’s estimate of sensitivity of 97.2% (*5*). Because antibody to nucleocapsid protein appears to decrease more rapidly than antibody to the spike protein, our analysis requires us to account for waning immunity (*27*,*28*). Our internal estimate of sensitivity is conditional on the distribution of time between infection and antibody testing for persons reported to be infected in our sample, which enhances its utility for adjusting the estimate of seroprevalence. Of note, among persons who reported having a prior test, 83% of serum samples were collected within 2 months following the previous RT-PCR SARS-CoV-2 test.

With these considerations in mind, our estimate of the detection fraction is substantially higher than what has been reported in other serologic surveys. A study that used residual clinical samples collected during March–May 2020 to measure SARS-CoV-2 antibody at 10 US sites estimated a detection fraction of 0.10 for residents of the country (*17*). That study estimated the seroprevalence in Utah at 2.2% (95% CI 1.2%–3.4%), and those CIs overlap with our estimate. Similarly, our estimate of seroprevalence is lower than what has been reported in most other geographic regions during a comparable period of the pandemic. In a meta-analysis that included 17 studies, the seroprevalence was estimated to be <1% in 5 of the studies examined (*29*). In another study, the projected prevalence of SARS-CoV-2 antibodies was 9.2% in the US adult population, based on an analysis of 28,000 dialysis patients; in Utah it was 3.1%. Discrepancies between results of other studies and our findings are likely due to our use of probabilistic sampling to reduce bias (*30*).

Our results suggest that Utah’s public health response to SARS-COV-2 was effective in case detection. Factors that likely contributed to the success of Utah’s approach to case detection include early expansion of access to testing, mobile testing that targeted heavily impacted communities, and a strong commitment to contact tracing and contact testing by the state and local health departments. This conclusion also is supported by our finding that 29% of seropositive persons reported exposure to a known case.

We observed that seropositivity was much more frequent than RT-PCR positivity, a finding that contrasts with selected other studies that combined viral detection and measurement of seroprevalence. For example, among randomly sampled residents of the US state of Indiana, the unadjusted prevalence of a positive RT-PCR was 1.74%, compared with an unadjusted SARS-CoV-2 seroprevalence of 1.01% (*31*). The ratio of prevalence of antibody detection to prevalence of viral detection, as observed in our community survey, suggests that infections were accumulating linearly rather than exponentially during the study period.

One limitation of our study is that it covers the early period of the COVID-19 pandemic, which reflects the cumulative incidence of SARS-CoV-2 infection through mid-June 2020. An updated analysis is needed to examine the secular trend in seroprevalence and determine whether the detection fraction continues to be high. Additional data also will enhance the feasibility of examining possible geographically localized hot spots. Our application of weighting and iterative proportional fitting should minimize nonresponse bias because of ethnicity and other measured factors at each stage of the sampling. However, our analytic approach cannot fully account for all sources of bias, particularly due to unmeasured factors that influenced the decision to participate at the household level. Thus, despite weighting techniques, the generalizability of our results might be limited by residual bias due to nonresponse. Nonetheless, our sampling frame likely reflects population seroprevalence more accurately than convenience-based samples. Recruitment efforts should focus on increasing the ease and appeal of participation of a wide range of demographic and geographic groups, especially for populations that traditionally have lower response rates and have been disproportionately affected by the COVID-19 pandemic.

In conclusion, we used a project design in which we randomly selected all participants, detected SARS-CoV-2 antibodies with a highly specific assay, applied rigorous analytical methods to account for bias and test error, and analyzed survey responses to support population-level inferences. The most distinctive finding in our analysis was that the detection fraction was estimated to be 40%. Further analysis is needed to determine whether this pattern has continued in subsequent months of the COVID-19 pandemic and to assess the factors that influence SARS-CoV-2 transmission and detection. High rates of testing and enhanced case detection are key initial steps for effective public health response.

This article was preprinted at https://www.medrxiv.org/content/10.1101/2020.10.26.20219907v1.

Appendix Additional information from this study of severe acute respiratory syndrome coronavirus 2 seroprevalence, Utah, USA.
