# Supplementary material for: Probability-Based Estimates of Severe Acute Respiratory Syndrome Coronavirus 2 Seroprevalence and Detection Fraction, Utah, USA
Source: Emerg Infect Dis. 2021 Nov;27(11):2786–94. doi: 10.3201/eid2711.204435 (PMC8544980; doi:10.3201/eid2711.204435)
Supplement: Appendix — Additional information from this study of severe acute respiratory syndrome coronavirus 2 seroprevalence, Utah, USA. [file 20-4435-Techapp-s1.pdf]

Article DOI: https://doi.org/10.3201/eid2711.204435

Probability-Based Estimates of Severe Acute Respiratory Syndrome Coronavirus 2 Seroprevalence and Detection Fraction, Utah, United States

Appendix

Statistical Methods

Complex Survey Design and Assay Error

Our statistical analyses incorporated several steps to account for nonresponse, demographic balance, and the sensitivity and specificity of the serology assay. We describe these steps below.

Step 1: Accounting for the sampling design. We estimated the probability (Pr) that a household was sampled in the primary sampling design as

Pr[household sampled] = Pr[tractgroup sampled] × ([no. of households sampled in tractgroup]/[no. viable addresses in tractgroup])

In strata for which ≥1 tractgroup was sampled, we approximated the probability that a given tractgroup was sampled as the product of the number of tractgroups sampled in that stratum and the probability of selection on a single draw. In the secondary sampling design, we approximated the probability that a household was targeted for sampling as the proportion of viable households within each stratum that were designated for sampling.

Step 2: Accounting for nonresponse. We estimated probabilities of response based on propensity models that used available information at the household, participant, and serology testing levels. We fit the propensity models separately for the primary and secondary sampling designs by using predictor variables (Appendix Table 1). We used boosted regression as implemented in the R TWANG statistical package (*1*) to estimate the propensities for a sampled household to respond to the household survey and for a survey respondent to provide serology

samples. We used logistic regression to estimate the propensities for participants to provide survey results among responding households. We computed weights to adjusted for overall nonresponse to serology testing as follows:

$$SW_{CIA1}(i) = \left\{\frac{1}{(Pr(HH\ sampled) \times (Pr\ (HH\ responds|HH\ sampled)) \times (Pr\ (Ind\ Responds|HH\ responds) \times (Pr(Ind\ Serology\ test|Ind\ Responds))}\right\}$$

where Pr(*HH sampled*) represents the sampling design probabilities for each household, Pr(*HH responds|HH sampled*) represents propensity score for household response, and Pr(*Ind Responds|HH responds*) represents propensity score for participant response, and Pr(*Ind serology test|Ind responds*) represents propensity score for serology test response (*2*).

Step 3: Aligning secondary sampling design to the primary sampling design. The primary sampling design included both mail-push-to-web survey and in-person interviews, providing a duplicative contact strategy with 2 modes of contact, whereas the secondary sampling design includes only the mail-push-to-web survey. Thus, we considered the primary sampling design to be less susceptible to nonresponse bias than the secondary sampling design. Therefore, we estimated a further set of propensity scores to reweight the participants providing serology samples in the secondary sampling frame to align the characteristics of the of the secondary sampling design to the primary sampling design. The propensity scores defining these weights also were estimated by using boosted regression and the following predictor variables obtained from participant responses to the survey: sex age; Hispanic ethnicity; education; believes social distancing is important; works outside the home at least a few times per week; level of COVID-19 concern; self-reported general health; self-report of being sick since March 1, 2020; and known contact with someone who with diagnosed COVID-19.

After obtaining propensity scores, we computed inverse probability of treatment weights to estimate average treatment affect in the treated (ATT) by using the following formula for each participant who provided a serum sample:

$$W^{P}_{CIAi} = \frac{Z_i}{1} + e_{CIAi}\frac{1-Z_i}{1-e_{CIAi}}$$

where Z_i, the “treatment”, indicates membership in the primary sampling design. We then updated the sampling weights by using the following formula:

$$SW_{CIA2}(i) = SW_{CIA1}(i) \times W^{P}_{CIAi}$$

Step 4: Averaging weights across sampling designs. We treated the weighted samples from the primary and secondary sampling designs as both representing the same population. Then we computed the weighted average of the weights across the primary and secondary designs based on the proportion of respondents from each individual sampling design relative to the total number of respondents.

Step 5: Weight trimming. We implemented weight trimming to reduce the variability in the sampling weights separately in each county (*3*). Weights that were <10% of the median weight were increased to 10% of the median, and weights that exceeded the median weight by a factor >10 were reduced to 10× the median.

Step 6: Iterative proportional fitting. Because nonresponse adjustments are limited to variables known at each step, imbalances in known characteristics might still differ between the sample and target population, even after applying the nonresponse weighs. Hence, we applied an additional calibration step by implementing iterative proportional fitting, often referred to as raking, to align the marginal distributions of age, sex, Hispanic ethnicity, and education between the weighted study sample and the population of the 4-county target population (*4*). We derived the population marginal distributions by using the 2018 Census American Community Survey 5-year estimates (*5*). The raking step was implemented using the following categorizations: age, categorized as 12–29, 30–59, or ≥60 years, by county; sex, categorized as male or female, by county; ethnicity, categorized as Hispanic versus non-Hispanic, by county but Davis County, due to insufficient sample size, was collapsed with Salt Lake County; education, categorized as completing 4-year college versus all others (including those <25 years of age), by county.

Strata and Primary Sampling Units

In addition to incorporating the appropriate weights, statistical analyses must also account for the strata within each sampling design and clustering of outcomes between different participants in the same primary sampling units (PSUs) within the same stratum. The information on the amount of variation in seroprevalence between the census tract groups, the true PSUs of the primary sampling design, was limited, because the primary sampling design had only 26 census tract groups across 15 strata, and 6/15 strata included only a single tract group. Possibly as a consequence of this limitation, variation in the estimated prevalence across the 26 tract groups within strata was less than expected by chance, preventing estimation of a clustering

effect. Therefore, we used the 54 census tracts rather than the census tract groups as the PSUs for the primary sampling design. For data analysis we also combined age strata among Salt Lake County low-prevalence Hispanic population; we also combined age strata among Salt Lake County low-prevalence non-Hispanic population due to insufficient census tracts within the individual strata. For the secondary sampling design, we used the more numerous block groups as the PSU in statistical analyses for all strata in which block groups were the true PSUs. For Park City, the used the household as the PSU in the secondary sampling design, and thus the household itself served as the PSU in data analysis.

Data Analysis

We constructed jackknife replicate weights (*6*), which we then applied in statistical analyses to estimate standard errors and perform statistical inference. Jackknife provides a largely model-free approach for estimating variability while accounting for correlations in outcomes between respondents in the same PSU, and naturally accounts for the use of different PSUs in the primary and secondary sampling designs. We modeled the relationship of seroprevalence and other outcomes, such as self-reported COVID-19 concern and self-reported social distancing, to predictor variables, such as county, demographic, and clinical factors, and behaviors and attitudes, by using survey-weighted generalized linear models for a binary outcome and assessed variability based on the replicate jackknife weights. We implemented these analyses by using the Survey package of R (R Foundation for Statistical Computing, https://www.r-project.org). We tested for the presence of a detectable temporal trend in seroprevalence by including calendar time as a continuous variable in models relating seroprevalence to the Utah Department of Health May 20, 2020 case count and calendar time. These analyses showed no trend for an effect of calendar time. Hence, analyses for seroprevalence are presented without adjustment for a secular trend in calendar time.

Adjusting Estimates of Seropositivity for Assay Error

Direct estimates of seroprevalence based on the proportion of tested respondents with positive serology assays are biased because the sensitivity and specificity of the test is expected to be <100%. Given relatively low seroprevalence, estimates of seroprevalence are especially strongly affected by the specificity of the test. As recommended by the Abbott Architect SARS-CoV-2 IgG package insert (*7*,*8*), we estimated specificity as 0.996, based on an evaluation of

1,070 samples obtained before the COVID-19 outbreak, including 73 samples from persons with other respiratory illnesses. This evaluation found that the assay incorrectly classified 4 of these 1,070 “true negative” samples as positive for COVID-19. We estimated a sensitivity of 0.83 which corresponded to the 25/30 respondents who reported having had a positive COVID-19 diagnosis and whose serology results were obtained ≥1 week later and were also positive. In sensitivity analyses we also considered a sensitivity estimate of 0.972, which is the proportion of 107 samples from subjects known to have COVID-19 that led to positive test results (104/107). These 107 samples included 73 from subjects with onset of COVID-19 symptoms at least 14 days before the test, and 34 subjects whose onset of COVID-19 symptoms was between 8 and 13 days before the test. Given these estimates of sensitivity and specificity, we then provided corrected estimates of seroprevalence by applying the formula (P1 – (1 – specificity))/(sensitivity + specificity – 1), where P1 is the estimated prevalence provided as described above. Finally, we used a parametric bootstrap resampling approach to account for the sampling error in the Abbott estimate of specificity when presenting lower and upper 95% confidence limits.

We did not further expand confidence limits to account for uncertainty in sensitivity. Instead, we conducted sensitivity analyses that evaluated how our estimates of seroprevalences are modified under different assumed values for the true sensitivity, which are compatible with the previous studies described in the prior paragraph.

References

1. Ridgeway G, McCaffrey D, Morral A, Burgette L, Griffin BA. Toolkit for weighting and analysis of nonequivalent groups: a tutorial for the twang package. Santa Monica, CA: RAND Corporation; 2017.

2. Valliant R, Dever JA, Kreuter F. Practical tools for designing and weighting survey samples. New York: Springer; 2013.

3. Lumley T. Complex surveys: a guide to analysis using R, volume 565. Hoboken (New Jersey, USA): John Wiley & Sons; 2011.

4. Lumley T. Post-stratification, raking and calibration. In: Complex surveys: a guide to analysis using R, volume 565. Hoboken (New Jersey, USA): John Wiley & Sons; 2011. p. 135–56.

5. US Census Bureau. American community survey, 2018: American community survey 5-year estimates [cited 2020 Aug 22]. https://data.census.gov/cedsci

6. Shao J, Tu D. The jackknife and bootstrap. New York: Springer-Verlag Inc; 1995.

7. Abbott Laboratories Diagnostics Division. SARS-CoV-2 IgG reagent kit for use with Architect, June 2020 [package insert]. [cited 2021 May 3]. https://www.fda.gov/media/137383/download

8. Abbott Laboratories Diagnostics Division. SARS-CoV-2 IgG calibrator kit for use with Architect, April 2020 [package insert] [cited 2021 May 3]. https://www.henryschein.com/assets/Medical/Abbott%20IFU%20-%20Calibration.pdf

Appendix Table 1. Predictor variables in propensity score nonresponse models used in a study of SARS-CoV-2 seroprevalence, Utah, United States*

Models and predictors
Household response propensity model
Location Predictors
1A) Tract group (primary sampling design only)
1B) Serology testing location (secondary sampling design only)
Predictors from U.S. Census
1) % of the population ≥14 y of age
2) Median age
3) % Hispanic
4) % not entering college
5) % of families with annual income <$60,000
6) % of families with annual income <$40,000
Individual response propensity model
Location Predictors
1A) Tract group (primary sampling design only)
1B) Serology testing location (secondary sampling design only)
Predictors from U.S. Census
1) % of families with annual income <$40,000
Predictors from household survey
1) Implements social distancing
2) Degree of concern over COVID-19
3) Regularly leaves the home for work, medical treatment, groceries, or to go to restaurants
4) General health
5) Hispanic ethnicity
6) Education level
7) Has been tested previously for COVID-19
8) Degree of concern that others should social distance
Serology response propensity model
Location Predictors
1A) Tract group (primary sampling design only)
1B) Serology testing location (secondary sampling design only)
Predictors from individual survey
1) Implements social distancing
2) Degree of concern over COVID-19
3) Regularly leaves the home for work, medical treatment, groceries, or to go to restaurants
4) General health
5) Respondent’s age
6) Respondent’s sex
7) Hispanic ethnicity
8) Education level
9) Has been tested previously for COVID-19
10) Degree of concern that others should social distance

*COVID-19, coronavirus disease; SARS-CoV-2, severe acute respiratory syndrome coronavirus 2.

Appendix Table 2. Household response rates in a study of SARS-CoV-2 seroprevalence, Utah, United States*

	Primary sampling design‡			Secondary sampling design§		
Stratum†	No. responded	No. approached	Response rate, %	No. responded	No. approached	Response rate, %
Davis County						
High prevalence	375	833	45	–	–	–
Low prevalence	374	1,036	36.1	–	–	–
High-low	–	–	–	274	2,125	12.9
Salt Lake County						
High prevalence						
Hispanic old	364	1,316	27.7	–	–	–
Hispanic young	210	834	25.2	–	–	–
Hispanic young and old	–	–	–	186	2,280	8.2
Non-Hispanic old	283	868	32.6	135	912	14.8
Non-Hispanic young	289	876	33	49	462	10.6
Low prevalence						
Hispanic old	131	415	31.6	36	456	7.9
Hispanic young	160	412	38.8	33	462	7.1
Non-Hispanic old	471	1,225	38.4	146	912	16
Non-Hispanic young	157	406	38.7	45	462	9.7
Summit County	165	876	18.8	118	3,205	3.7
Utah County						
High prevalence						
Hispanic	258	818	31.5	88	912	9.6
Non-Hispanic	146	416	35.1	47	456	10.3
Low prevalence						
Hispanic	161	411	39.2	–	–	–
Non-Hispanic	294	821	35.8	–	–	–
Hispanic and non-Hispanic	–	–	–	130	1,368	9.5

*SARS-CoV-2, severe acute respiratory syndrome coronavirus 2.
†Prevalence refers to the incidence proportion of SARS-COV-2 infection based on reported SARS-CoV-2 case counts at the time that data collection began.
‡In the primary sampling design, we operationally defined household approaches as a visit by the field team or the initiation of the online survey in response to the mailer sent to the household. Respondents were households that completed key portions of the household survey or ≥1 individual survey. We estimated the response rates as the ratio of these 2 quantities.
§In the secondary sampling design, we defined household approaches as households being sent the mailer. We used different definitions between the 2 sampling designs because the principal sampling method in the primary sampling design was door-to-door contact by the field team, with mailings playing a secondary role, while in the secondary sampling design the only sampling method was the mailer.

Appendix Table 3. Participant response rates for surveys in 4 counties in a study of SARS-CoV-2 seroprevalence, Utah, United States*

	Primary sampling design‡			Secondary sampling design§		
Stratum†	No. responded	No. approached	Response rate, %	No. responded	No. approached	Response rate, %
Davis County						
High prevalence	741	950	78	–	–	–
Low prevalence	764	1,100	69.5	–	–	–
High-low prevalence	–	–	–	576	697	82.6
Salt Lake County						
High prevalence						
Hispanic old	614	774	79.3	–	–	–
Hispanic young	325	505	64.4	–	–	–
Hispanic young and old	–	–	–	348	404	86.1
Non-Hispanic old	518	639	81.1	275	315	87.3
Non-Hispanic young	471	590	79.8	96	107	89.7
Low prevalence						
Hispanic old	258	340	75.9	69	82	84.1
Hispanic young	314	457	68.7	69	83	83.1
Non-Hispanic old	908	1233	73.6	314	354	88.7
Non-Hispanic young	340	514	66.1	92	99	92.9
Summit County	160	177	90.4	171	202	84.7
Utah County						
High prevalence						
Hispanic	524	706	74.2	195	234	83.3
Non-Hispanic	305	413	73.8	124	147	84.4
Low prevalence						
Hispanic	352	532	66.2	–	–	–
Non-Hispanic	598	843	70.9	–	–	–
Hispanic and non-Hispanic	–	–	–	312	378	82.5

*We defined individual response rates in both sampling designs as the proportion of persons ≥12 years of age in responding households that completed the survey. SARS-CoV-2, severe acute respiratory syndrome coronavirus 2.

†Prevalence refers to the incidence proportion of SARS-COV-2 infection based on reported SARS-CoV-2 case counts at the time that data collection began.

‡In the primary sampling design, we operationally defined household approaches as a visit by the field team or the initiation of the online survey in response to the mailer sent to the household. Respondents were households that completed key portions of the household survey or ≥1 individual survey. We estimated the response rates as the ratio of these 2 quantities.

§In the secondary sampling design, we defined household approaches as households being sent the mailer. We used different definitions between the 2 sampling designs because the principal sampling method in the primary sampling design was door-to-door contact by the field team, with mailings playing a secondary role, while in the secondary sampling design the only sampling method was the mailer.

Appendix Table 4. Serology response rates in 4 counties in a study of SARS-CoV-2 seroprevalence, Utah, United States*

	Primary sampling design‡			Secondary sampling design§		
Stratum†	No. responded	No. approached	Response rate, %	No. responded	No. approached	Response rate, %
Davis County						
High prevalence	593	746	79.5	–	–	–
Low prevalence	594	791	75.1	–	–	–
High-low prevalence	–	–	–	516	598	86.3
Salt Lake County						
High prevalence						
Hispanic old	512	651	78.6	–	–	–
Hispanic young	201	348	57.8	–	–	–
Hispanic young and old	–	–	–	287	361	79.5
Non-Hispanic old	429	534	80.3	245	289	84.8
Non-Hispanic young	352	489	72	87	100	87
Low prevalence						
Hispanic old	217	272	79.8	63	69	91.3
Hispanic young	227	332	68.4	60	73	82.2
Non-Hispanic old	732	958	76.4	274	320	85.6
Non-Hispanic young	252	356	70.8	83	93	89.2
Summit County	218	277	78.7	127	179	70.9
Utah County						
High prevalence						
Hispanic	441	554	79.6	171	195	87.7
Non-Hispanic	261	329	79.3	124	141	87.9
Low prevalence						
Hispanic	288	363	79.3	–	–	–
Non-Hispanic	474	619	76.6	–	–	–
Hispanic and non-Hispanic	–	–	–	280	331	84.6

*We defined serology response rates in both sampling designs as the proportion of survey respondents who also provided a serology sample. SARS-CoV-2, severe acute respiratory syndrome coronavirus 2.

†Prevalence refers to the incidence proportion of SARS-COV-2 infection based on reported SARS-CoV-2 case counts at the time that data collection began.

‡In the primary sampling design, we operationally defined household approaches as a visit by the field team or the initiation of the online survey in response to the mailer sent to the household. Respondents were households that completed key portions of the household survey or ≥1 individual survey. We estimated the response rates as the ratio of these 2 quantities.

§In the secondary sampling design, we defined household approaches as households being sent the mailer. We used different definitions between the 2 sampling designs because the principal sampling method in the primary sampling design was door-to-door contact by the field team, with mailings playing a secondary role, while in the secondary sampling design the only sampling method was the mailer.

Appendix Table 5. Overall response rates in 4 counties in a study of SARS-CoV-2 seroprevalence, Utah, United States*

Stratum†	Sampling design, % response	
	Primary‡	Secondary§
Davis County		
High prevalence	27.9	–
Low prevalence	18.8	–
High-low prevalence	–	9.2
Salt Lake County		
High prevalence		
Hispanic old	17.3	–
Hispanic young	9.4	–
Hispanic young and old	–	5.6
Non-Hispanic old	21.2	11
Non-Hispanic young	19	8.3
Low prevalence		
Hispanic old	19.1	6.1
Hispanic young	18.2	4.8
Non-Hispanic old	21.6	12.1
Non-Hispanic young	18.1	8
Summit County	13.4	2.2
Utah County		
High prevalence		
Hispanic	18.6	7
Non-Hispanic	20.5	7.6
Low prevalence		
Hispanic	20.6	–
Non-Hispanic	19.4	–
Hispanic and non-Hispanic	–	6.6

*We estimated overall response as the products of the household, individual, and serology level response rates from Appendix Tables 3, 4, and 5. SARS-CoV-2, severe acute respiratory syndrome coronavirus 2.

†Prevalence refers to the incidence proportion of SARS-COV-2 infection based on reported SARS-CoV-2 case counts at the time that data collection began.

‡In the primary sampling design, we operationally defined household approaches as a visit by the field team or the initiation of the online survey in response to the mailer sent to the household. Respondents were households that completed key portions of the household survey or ≥1 individual survey. We estimated the response rates as the ratio of these 2 quantities.

§In the secondary sampling design, we defined household approaches as households being sent the mailer. We used different definitions between the 2 sampling designs because the principal sampling method in the primary sampling design was door-to-door contact by the field team, with mailings playing a secondary role, while in the secondary sampling design the only sampling method was the mailer.

Appendix Table 6. Summaries of the mean relative weights applied to various subgroups of respondents in a study of SARS-CoV-2 seroprevalence, Utah, United States*

Variable	Sample size	Relative mean analysis weight†
Age group, y		
12–17	755	1.6
18–44	3,366	1.2
45–64	2,345	0.9
65–74	1,087	0.6
75–84	477	0.6
≥85	78	0.6
Sex		
M	3,773	1.1
F	4,293	0.9
Ethnicity		
Hispanic	528	2.3
Non-Hispanic	7,516	0.9
Education Level		
High school or less	1,681	1.7
Some college or technical school	2,022	1.4
College graduate	4,281	0.5
General Health		
Excellent	2,404	1
Very Good	3,443	1
Good	1,792	1
Fair/Poor	444	1
Chronic Medical Conditions		
None	5,567	1.1
Asthma only	841	1
≥1 chronic condition other than asthma	1,700	0.8

*The ratios of the mean analysis sampling weights within the designated subgroup compared with the overall mean sampling rate for analyses of the serology results are shown. These ratios indicate the relative amount of influence of individual respondents with different characteristics. The weights incorporate adjustments for nonresponses at the household, individual, and serology levels and the propensity score adjustment used to align the characteristics of respondents in the secondary sampling design to respondents in the primary sampling design and to the final iterative proportional fitting step to align the weighted characteristics of the study population to the U.S. census.

†Relative mean analysis weight = mean × (weights for subgroup)/mean × (weight for everyone), where weights are the final analysis weights that account for sampling design and all postsurvey adjustments.

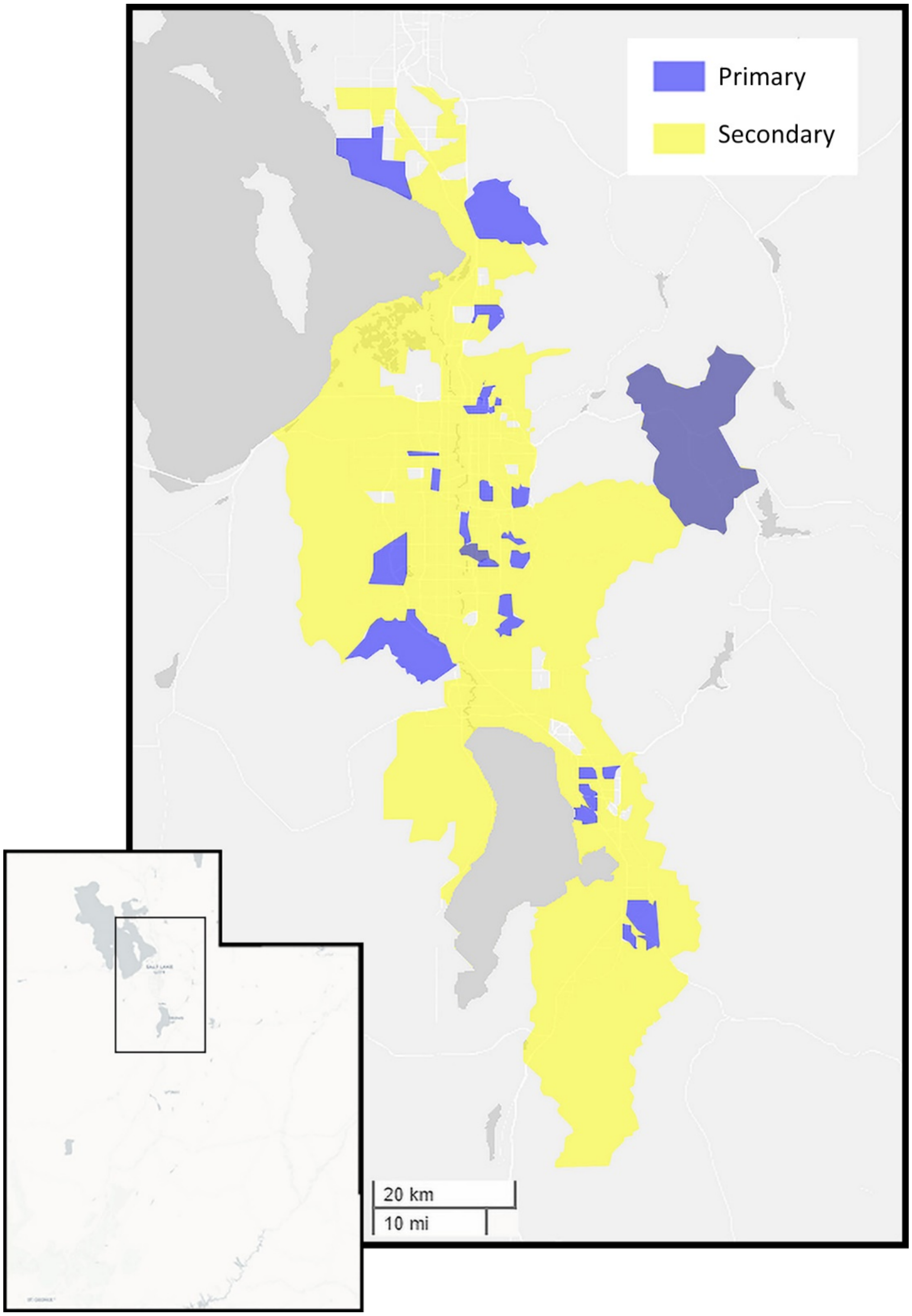

Appendix Figure 1. Geographic locations of the primary or secondary sampling designs in a study of SARS-CoV-2 seroprevalence, Utah, United States. The figure illustrates that the primary sampling locations are spread across 4 counties and that a large fraction of the counties were sampled either in the primary or secondary sampling design. Inset shows Utah with the 4-country area shown by box. SARS-CoV-2, severe acute respiratory syndrome coronavirus 2.

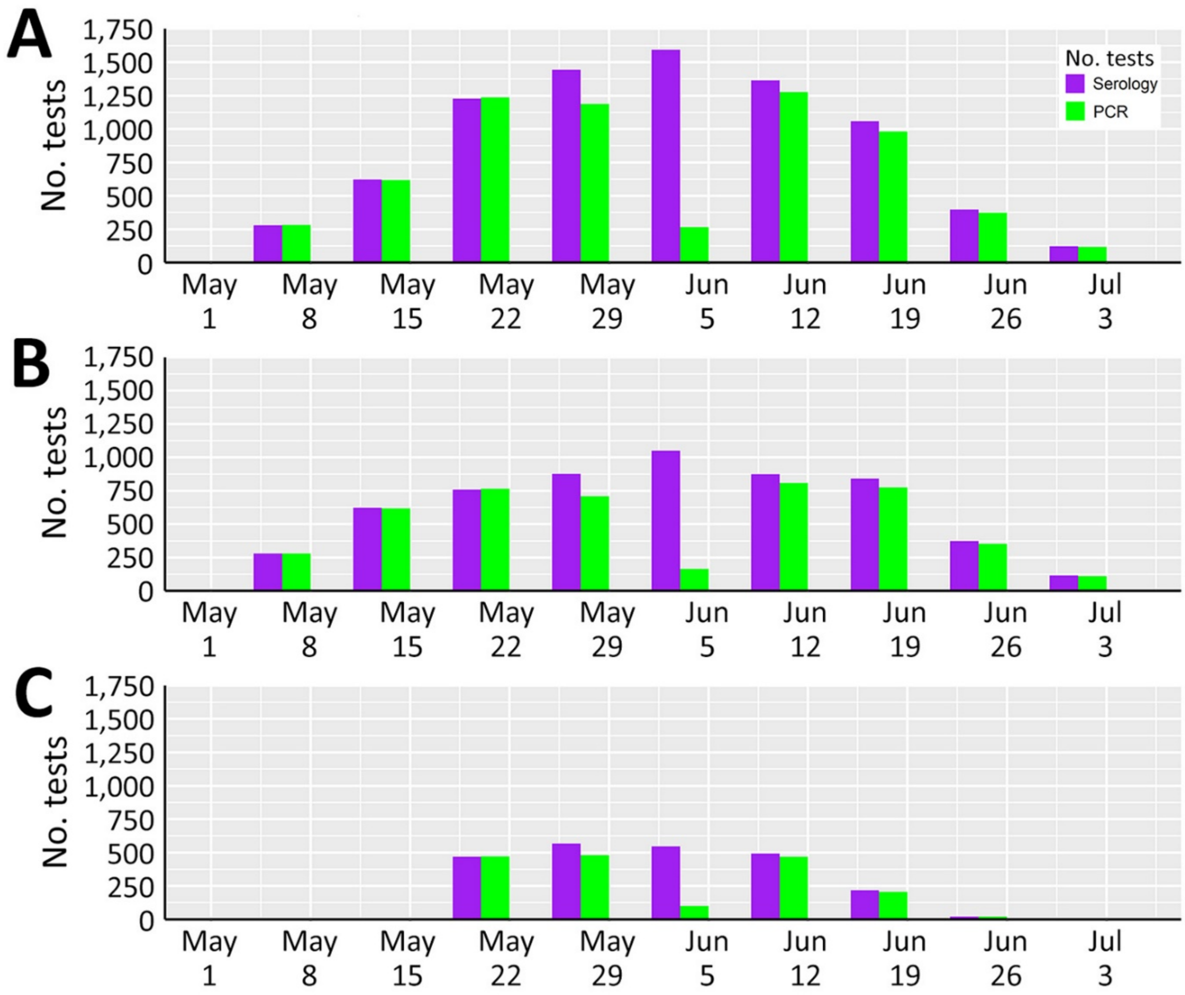

Appendix Figure 2. Timing of serology and PCR samples in a study of SARS-CoV-2 seroprevalence, Utah, United States. Top, extended sampling design; middle, primary sampling design; bottom, secondary sampling design. Extended sampling design refers to collection of all 5,125 responding households, including households in both the primary and secondary sampling designs. SARS-CoV-2, severe acute respiratory syndrome coronavirus 2.

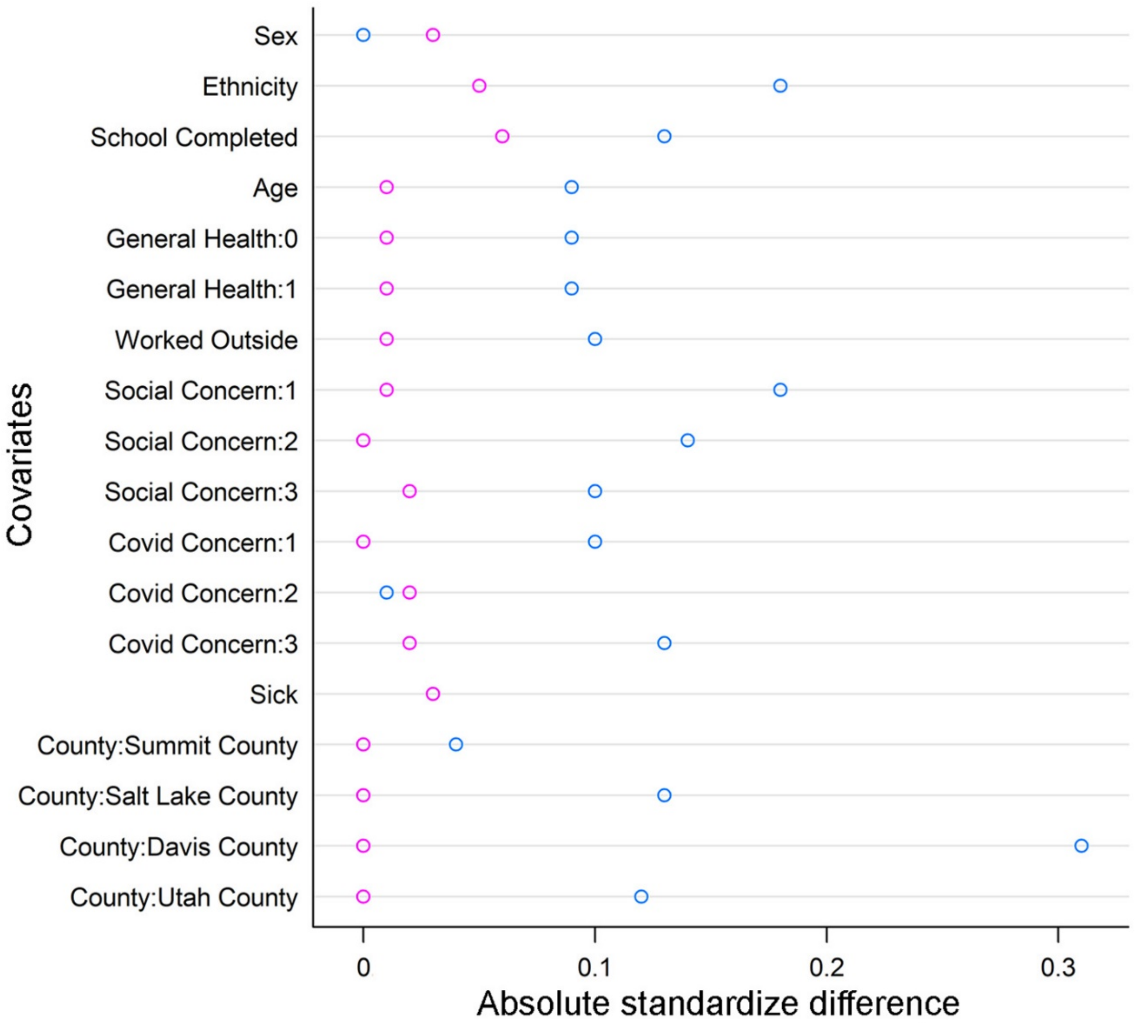

Appendix Figure 3. Propensity matching of secondary to primary sampling design respondents in a study of SARS-CoV-2 seroprevalence, Utah, United States. Blue circles represent the standardized mean differences in each factor between the primary and secondary sampling designs after application of sampling weights that account for nonresponse at the household, individual, and serology testing levels. Pink circles represent the standardized mean differences after the additional propensity score weighting to bring the characteristics of the respondents in the secondary sampling design into alignment with the characteristics of the respondents in the primary sampling design. The shift in the pink circles relative to the blue circles indicates the effect of the propensity adjustment to align the secondary design sample to match the primary design sample.

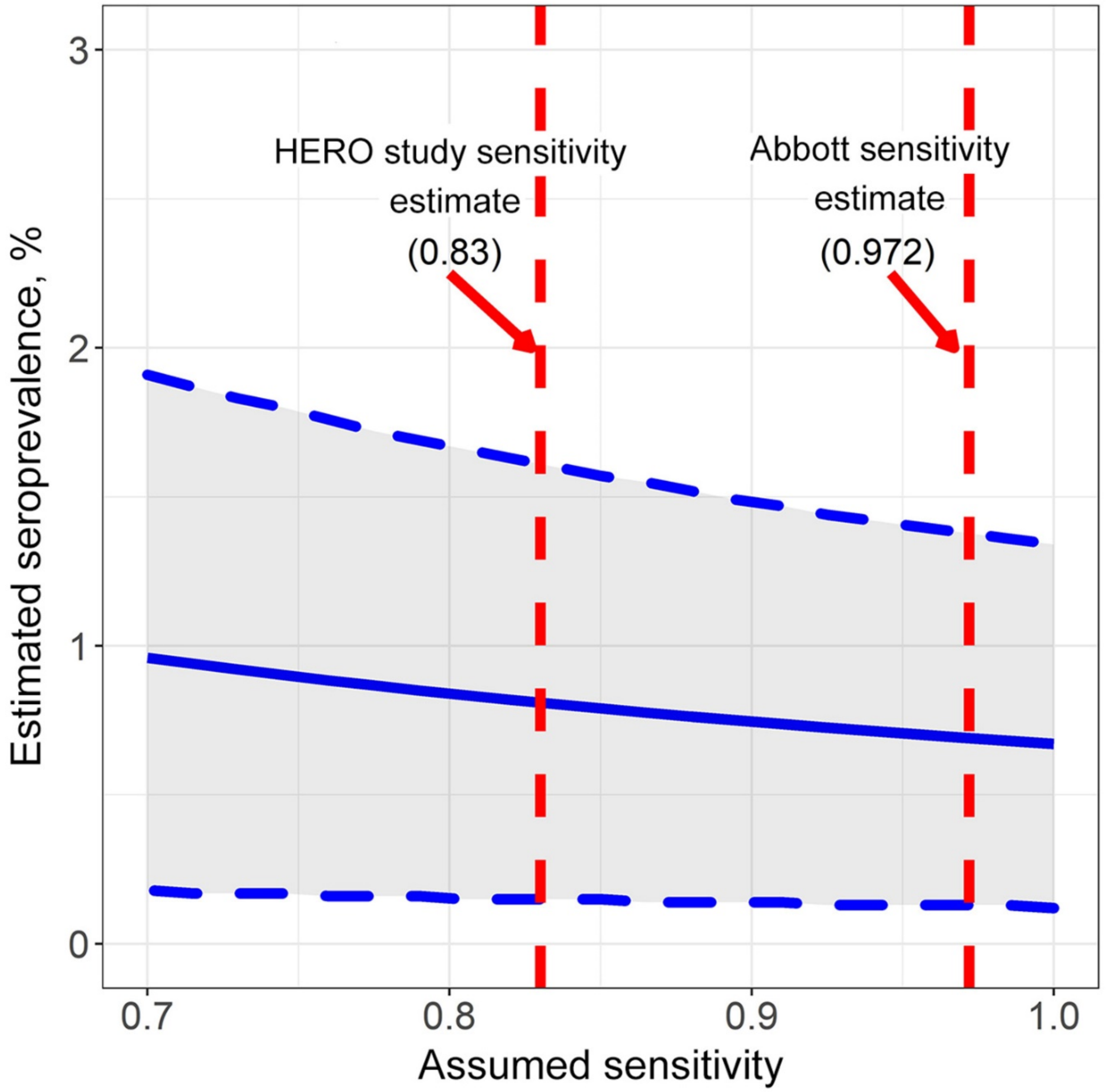

Appendix Figure 4. Dependence of percent seropositivity on assumed sensitivity of the serology assay used for analyses in a study of SARS-CoV-2 seroprevalence, Utah, United States. Our primary estimates of seroprevalence are based on estimates of 0.83 sensitivity, selected on the basis of the fraction of respondents (25/30) who self-reported having a prior positive COVID-19 test and subsequently had a positive serology test ≥1 week after their reported positive COVID-19 test. We considered a relatively wide range for sensitivity to address speculation that IgG concentrations might wane over time and become undetectable by the assay at some point. The graph shows the relationship between the estimated seroprevalence across the 4-county area with the assumed sensitivity if specificity is assumed to be 0.996. COVID-19, coronavirus 19; HERO, Health and Economic Recovery Outreach program in Utah; SARS-CoV-2, severe acute respiratory syndrome coronavirus 2.

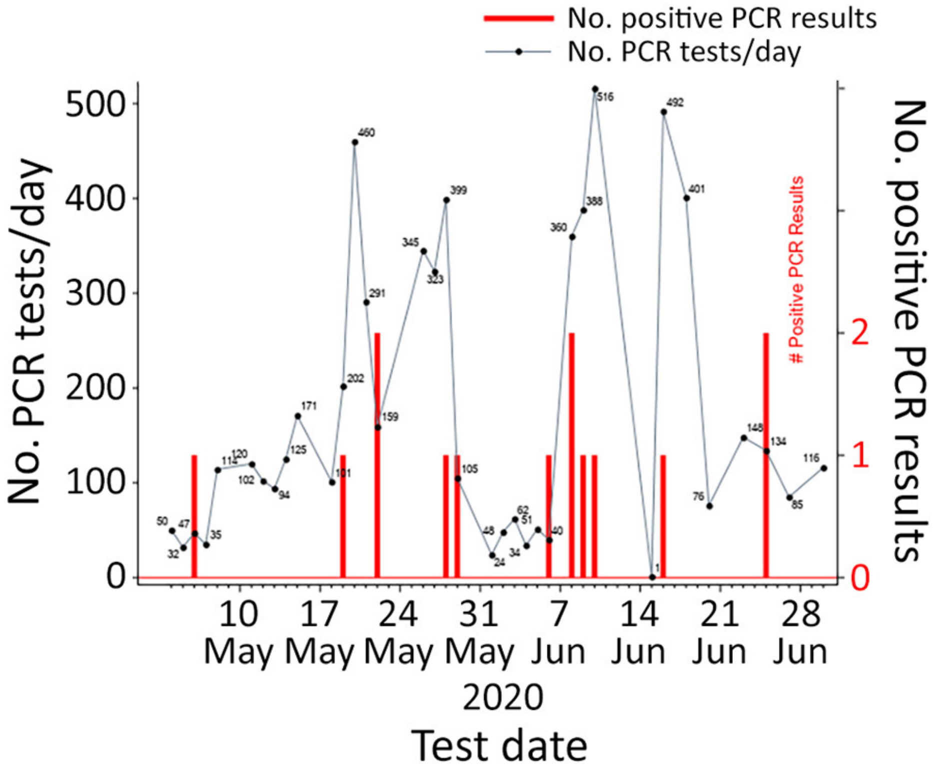

Appendix Figure 5. Positive PCR tests and total number of PCR tests performed on participants in a study of SARS-CoV-2 seroprevalence, Utah, United States. Gray curve indicates the number of PCR tests performed each day; red bars indicate number of PCR-positive results per week. The drop-off in the gray curve in late May and early June reflects a temporary period during which PCR tests were administered only when specifically requested by the respondent. The study subsequently reinitiated broad PCR testing in response to the increased COVID-19 case counts reported in the 4-county area. COVID-19, coronavirus 19; SARS-CoV-2, severe acute respiratory syndrome coronavirus 2.
